# Glycinium 3-carb­oxy-4-hy­droxy­benzene­sulfonate

**DOI:** 10.1107/S1600536814004590

**Published:** 2014-03-05

**Authors:** A. Thirunavukkarasu, A. Silambarasan, R. Mohan Kumar, P. R. Umarani, G. Chakkaravarthi

**Affiliations:** a Department of Physics, Presidency College, Chennai 600 005, India; b Kunthavai Naacchiyaar Govt. Arts College (W), Thanjavur 613 007, India; c Department of Physics, CPCL Polytechnic College, Chennai 600 068, India

## Abstract

In the anion of the title salt, C_2_H_6_NO_2_^+^·C_7_H_5_O_6_S^−^, the dihedral angle between the carb­oxy­lic acid group and the benzene ring is 5.02 (12)°. In the crystal, the anions are linked into inversion dimers through pairs of O—H⋯O hydrogen bonds between the carb­oxy­lic acid groups and sulfonate O atoms. A pair of C—H⋯O inter­actions is also observed within each dimer. The anion dimers and the cations are linked into a three-dimensional network by N—H⋯O, O—H⋯O and C—H⋯O hydrogen bonds.

## Related literature   

For background to non-linear optical materials, see: Yang *et al.* (2005 ▶); Kumar *et al.* (2009 ▶). For related structures, see: Krishnakumar *et al.* (2012 ▶); Sudhahar *et al.* (2013 ▶).
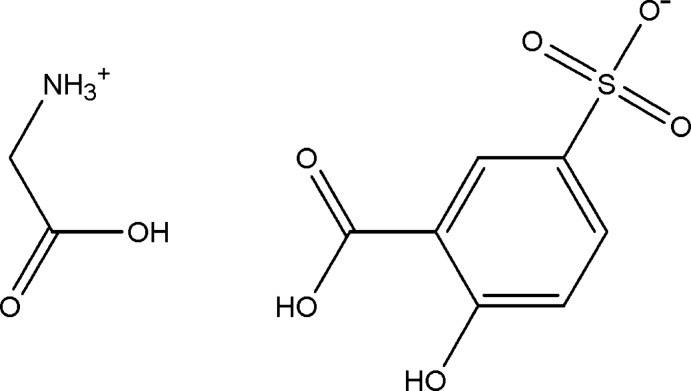


## Experimental   

### 

#### Crystal data  

C_2_H_6_NO_2_^+^·C_7_H_5_O_6_S^−^
*M* *_r_* = 293.25
Monoclinic, 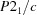

*a* = 5.3651 (3) Å
*b* = 24.7207 (15) Å
*c* = 8.6840 (5) Å
β = 90.170 (2)°
*V* = 1151.75 (12) Å^3^
*Z* = 4
Mo *K*α radiation
μ = 0.32 mm^−1^
*T* = 295 K
0.36 × 0.32 × 0.30 mm

#### Data collection  

Bruker Kappa APEXII CCD diffractometer
Absorption correction: multi-scan (*SADABS*; Sheldrick, 1996 ▶) *T*_min_ = 0.893, *T*_max_ = 0.910
21406 measured reflections
3694 independent reflections
3282 reflections with *I* > 2σ(*I*)
*R*_int_ = 0.030

#### Refinement  

*R*[*F* ^2^ > 2σ(*F* ^2^)] = 0.049
*wR*(*F* ^2^) = 0.117
*S* = 1.20
3694 reflections
197 parameters
6 restraints
H atoms treated by a mixture of independent and constrained refinement
Δρ_max_ = 0.49 e Å^−3^
Δρ_min_ = −0.53 e Å^−3^

### 

Data collection: *APEX2* (Bruker, 2004 ▶); cell refinement: *SAINT* (Bruker, 2004 ▶); data reduction: *SAINT*; program(s) used to solve structure: *SHELXS97* (Sheldrick, 2008 ▶); program(s) used to refine structure: *SHELXL97* (Sheldrick, 2008 ▶); molecular graphics: *PLATON* (Spek, 2009 ▶); software used to prepare material for publication: *SHELXL97*.

## Supplementary Material

Crystal structure: contains datablock(s) global, I. DOI: 10.1107/S1600536814004590/is5343sup1.cif

Structure factors: contains datablock(s) I. DOI: 10.1107/S1600536814004590/is5343Isup2.hkl

Supporting information file. DOI: 10.1107/S1600536814004590/is5343Isup3.cml

CCDC reference: 989106

Additional supporting information:  crystallographic information; 3D view; checkCIF report

## Figures and Tables

**Table 1:** Hydrogen-bond geometry (Å, °)

*D*—H⋯*A*	*D*—H	H⋯*A*	*D*⋯*A*	*D*—H⋯*A*
O1—H1⋯O6	0.82 (1)	1.89 (2)	2.631 (2)	150 (4)
N1—H1*A*⋯O6	0.86 (1)	2.27 (3)	2.878 (2)	127 (3)
N1—H1*A*⋯O7^i^	0.86 (1)	2.46 (3)	3.134 (2)	135 (3)
N1—H1*B*⋯O3^ii^	0.87 (1)	2.06 (2)	2.876 (2)	157 (3)
N1—H1*C*⋯O3^iii^	0.87 (1)	1.98 (2)	2.811 (2)	161 (3)
O5—H5*A*⋯O4^iv^	0.82 (1)	1.92 (2)	2.702 (2)	159 (3)
O7—H7⋯O2^v^	0.82 (1)	1.84 (1)	2.646 (2)	169 (3)
C2—H2⋯O5^iv^	0.93	2.45	3.370 (2)	168
C9—H9*A*⋯O4^iv^	0.97	2.33	3.292 (2)	172
C6—H6⋯O8^vi^	0.93	2.46	3.273 (2)	147
C9—H9*B*⋯O2^vii^	0.97	2.37	3.324 (2)	167

Symmetry codes: (i) 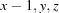; (ii) 
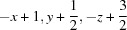
; (iii) 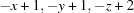; (iv) 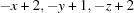; (v) 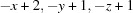; (vi) 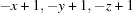; (vii) 
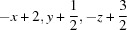
.

**Fig. 1.**
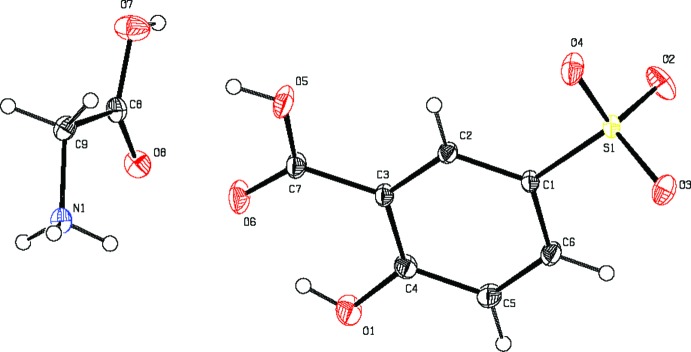
The molecular structure of the title compound, with atom labels and 30% probability displacement ellipsoids for non-H atoms.

**Fig. 2.**
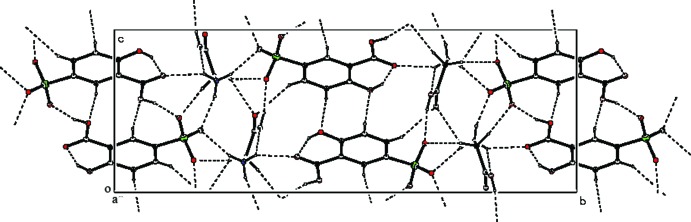
A packing diagram of the title compound, viewed down the a axis. Intermolecular hydrogen bonds are shown as dashed lines. H atoms not involved in hydrogen bonding have been omitted.

## supplementary crystallographic information

### 1. Comment

Non-linear optical materials have recently invoked a large amount of interest due to their potential application in harmonic generation, optical information processing, optical storage and two photon pumped lasers (Yang *et al.*, 2005; Kumar *et al.*, 2009). We herein, report the crystal structure of the title compound (I), (Fig. 1). The geometric parameters of the title compound are comparable with the reported structures (Krishnakumar *et al.*, 2012; Sudhahar *et al.*, 2013).

In the molecular structure, the cation and anion are linked by N—H···O and O—H···O hydrogen bonds. In the anion, the dihedral angle between the carboxyl group and the benzene ring is 5.02 (12)°. The crystal structure exhibits intermolecular N—H···O, O—H···O and C—H···O (Table 1 & Fig. 2) interactions.

### 2. Experimental

The title compound was obtained by slow evaporation from an aqueous solution of glycine (C_2_H_5_NO_2_, 0.75 g) and 3-carboxy-4-hydroxybenzenesulfonic acid (C_7_H_6_O_6_S, 2.18 g) at room temperature. The good quality crystals suitable for X-ray diffraction were collected in the period of 20 days.

### 3. Refinement

H atoms of the NH_3_ and OH groups were located in a difference Fourier map and refined freely, with bond-length restraints of N—H = 0.86 (1) Å and O—H = 0.82 (1) Å. The C-bound H atoms were positioned geometrically and refined using a riding model, with C—H = 0.93 and *U*_iso_(H) = 1.2*U*_eq_(C) for CH and C—H = 0.97 Å and *U*_iso_(H) = 1.2*U*_eq_(C) for CH_2_.

### Crystal data

**Table:**

C_2_H_6_NO_2_^+^·C_7_H_5_O_6_S^−^	*F*(000) = 608
*M**_r_* = 293.25	*D*_x_ = 1.691 Mg m^−^^3^
Monoclinic, *P*2_1_/*c*	Mo *K*α radiation, λ = 0.71073 Å
Hall symbol: -P 2ybc	Cell parameters from 9634 reflections
*a* = 5.3651 (3) Å	θ = 2.5–32.0°
*b* = 24.7207 (15) Å	µ = 0.32 mm^−^^1^
*c* = 8.6840 (5) Å	*T* = 295 K
β = 90.170 (2)°	Block, colourless
*V* = 1151.75 (12) Å^3^	0.36 × 0.32 × 0.30 mm
*Z* = 4	

### Data collection

**Table:**

Bruker Kappa APEXII CCD diffractometer	3694 independent reflections
Radiation source: fine-focus sealed tube	3282 reflections with *I* > 2σ(*I*)
Graphite monochromator	*R*_int_ = 0.030
ω and φ scan	θ_max_ = 32.1°, θ_min_ = 1.7°
Absorption correction: multi-scan (*SADABS*; Sheldrick, 1996)	*h* = −7→7
*T*_min_ = 0.893, *T*_max_ = 0.910	*k* = −36→36
21406 measured reflections	*l* = −12→12

### Refinement

**Table:**

Refinement on *F*^2^	Secondary atom site location: difference Fourier map
Least-squares matrix: full	Hydrogen site location: inferred from neighbouring sites
*R*[*F*^2^ > 2σ(*F*^2^)] = 0.049	H atoms treated by a mixture of independent and constrained refinement
*wR*(*F*^2^) = 0.117	*w* = 1/[σ^2^(*F*_o_^2^) + (0.0325*P*)^2^ + 1.2564*P*] where *P* = (*F*_o_^2^ + 2*F*_c_^2^)/3
*S* = 1.20	(Δ/σ)_max_ < 0.001
3694 reflections	Δρ_max_ = 0.49 e Å^−^^3^
197 parameters	Δρ_min_ = −0.53 e Å^−^^3^
6 restraints	Extinction correction: *SHELXL97* (Sheldrick, 2008), Fc^*^=kFc[1+0.001xFc^2^λ^3^/sin(2θ)]^-1/4^
Primary atom site location: structure-invariant direct methods	Extinction coefficient: 0.071 (3)

### Fractional atomic coordinates and isotropic or equivalent isotropic displacement parameters (Å^2^)

**Table:**

	*x*	*y*	*z*	*U*_iso_*/*U*_eq_	
C1	0.5636 (3)	0.41186 (7)	0.7824 (2)	0.0177 (3)	
C2	0.6669 (3)	0.46042 (7)	0.8273 (2)	0.0194 (3)	
H2	0.8074	0.4607	0.8903	0.023*	
C3	0.5608 (3)	0.50936 (7)	0.7784 (2)	0.0198 (3)	
C4	0.3495 (4)	0.50846 (8)	0.6831 (2)	0.0244 (4)	
C5	0.2445 (4)	0.45897 (8)	0.6403 (3)	0.0283 (4)	
H5	0.1027	0.4583	0.5785	0.034*	
C6	0.3505 (4)	0.41112 (8)	0.6893 (2)	0.0244 (4)	
H6	0.2801	0.3783	0.6603	0.029*	
C7	0.6683 (4)	0.56133 (7)	0.8269 (2)	0.0248 (4)	
C8	0.8846 (4)	0.69748 (7)	0.6240 (2)	0.0213 (3)	
C9	0.8379 (4)	0.71637 (8)	0.7865 (2)	0.0221 (3)	
H9A	0.9096	0.6908	0.8589	0.026*	
H9B	0.9166	0.7512	0.8028	0.026*	
N1	0.5669 (3)	0.72092 (7)	0.81273 (19)	0.0219 (3)	
O1	0.2384 (3)	0.55374 (7)	0.6301 (2)	0.0398 (4)	
O2	0.8214 (3)	0.32854 (7)	0.69766 (18)	0.0377 (4)	
O3	0.5004 (3)	0.31390 (6)	0.88206 (18)	0.0305 (3)	
O4	0.8755 (3)	0.36047 (6)	0.9590 (2)	0.0364 (4)	
O5	0.8540 (3)	0.55664 (6)	0.9256 (2)	0.0364 (4)	
O6	0.5921 (3)	0.60513 (6)	0.7813 (2)	0.0380 (4)	
O7	1.1214 (3)	0.68486 (8)	0.60241 (19)	0.0386 (4)	
O8	0.7240 (3)	0.69592 (6)	0.52779 (16)	0.0278 (3)	
S1	0.70129 (8)	0.349884 (16)	0.83495 (5)	0.01747 (12)	
H1A	0.489 (6)	0.6942 (10)	0.771 (4)	0.059 (10)*	
H1B	0.515 (6)	0.7512 (8)	0.774 (4)	0.059 (10)*	
H1C	0.528 (6)	0.7171 (14)	0.9088 (15)	0.059 (10)*	
H5A	0.910 (6)	0.5865 (7)	0.949 (4)	0.051 (9)*	
H7	1.134 (6)	0.6766 (12)	0.5112 (15)	0.048 (9)*	
H1	0.320 (6)	0.5791 (10)	0.665 (4)	0.064 (11)*	

### Atomic displacement parameters (Å^2^)

**Table:**

	*U*^11^	*U*^22^	*U*^33^	*U*^12^	*U*^13^	*U*^23^
C1	0.0206 (8)	0.0139 (7)	0.0186 (8)	−0.0001 (6)	−0.0015 (6)	−0.0008 (6)
C2	0.0198 (8)	0.0163 (7)	0.0219 (8)	−0.0006 (6)	−0.0050 (6)	−0.0003 (6)
C3	0.0229 (8)	0.0147 (7)	0.0218 (8)	−0.0006 (6)	−0.0037 (6)	0.0001 (6)
C4	0.0261 (9)	0.0201 (8)	0.0270 (9)	0.0030 (7)	−0.0065 (7)	0.0031 (7)
C5	0.0249 (9)	0.0258 (9)	0.0342 (11)	0.0013 (7)	−0.0141 (8)	−0.0010 (8)
C6	0.0249 (9)	0.0196 (8)	0.0286 (9)	−0.0025 (6)	−0.0071 (7)	−0.0034 (7)
C7	0.0292 (10)	0.0161 (7)	0.0291 (9)	−0.0014 (6)	−0.0055 (8)	0.0003 (7)
C8	0.0271 (9)	0.0181 (7)	0.0186 (8)	−0.0005 (6)	0.0015 (7)	0.0008 (6)
C9	0.0255 (9)	0.0238 (8)	0.0169 (8)	−0.0034 (7)	−0.0012 (6)	−0.0017 (6)
N1	0.0278 (8)	0.0198 (7)	0.0181 (7)	−0.0003 (6)	0.0008 (6)	−0.0003 (6)
O1	0.0426 (9)	0.0232 (7)	0.0534 (11)	0.0065 (7)	−0.0235 (8)	0.0052 (7)
O2	0.0510 (10)	0.0343 (8)	0.0279 (8)	0.0189 (7)	0.0124 (7)	0.0003 (6)
O3	0.0374 (8)	0.0217 (6)	0.0324 (8)	−0.0097 (6)	0.0019 (6)	0.0069 (6)
O4	0.0463 (9)	0.0209 (7)	0.0419 (9)	0.0003 (6)	−0.0261 (8)	−0.0003 (6)
O5	0.0452 (9)	0.0190 (7)	0.0449 (9)	−0.0059 (6)	−0.0232 (8)	−0.0013 (6)
O6	0.0492 (10)	0.0150 (6)	0.0496 (10)	0.0014 (6)	−0.0166 (8)	0.0022 (6)
O7	0.0282 (8)	0.0602 (11)	0.0274 (8)	0.0057 (7)	0.0009 (6)	−0.0083 (7)
O8	0.0328 (8)	0.0322 (7)	0.0183 (6)	0.0033 (6)	−0.0035 (5)	−0.0020 (5)
S1	0.0230 (2)	0.01263 (18)	0.0168 (2)	−0.00042 (14)	−0.00211 (14)	−0.00052 (13)

### Geometric parameters (Å, º)

**Table:**

C1—C2	1.378 (2)	C8—O7	1.322 (2)
C1—C6	1.399 (3)	C8—C9	1.508 (3)
C1—S1	1.7605 (17)	C9—N1	1.477 (3)
C2—C3	1.402 (2)	C9—H9A	0.9700
C2—H2	0.9300	C9—H9B	0.9700
C3—C4	1.402 (3)	N1—H1A	0.863 (10)
C3—C7	1.470 (2)	N1—H1B	0.866 (10)
C4—O1	1.348 (2)	N1—H1C	0.865 (10)
C4—C5	1.397 (3)	O1—H1	0.823 (10)
C5—C6	1.379 (3)	O2—S1	1.4559 (15)
C5—H5	0.9300	O3—S1	1.4570 (14)
C6—H6	0.9300	O4—S1	1.4478 (15)
C7—O6	1.223 (2)	O5—H5A	0.822 (10)
C7—O5	1.317 (2)	O7—H7	0.821 (10)
C8—O8	1.199 (2)		
			
C2—C1—C6	120.16 (16)	O7—C8—C9	111.59 (17)
C2—C1—S1	121.11 (13)	N1—C9—C8	109.53 (15)
C6—C1—S1	118.69 (13)	N1—C9—H9A	109.8
C1—C2—C3	120.21 (16)	C8—C9—H9A	109.8
C1—C2—H2	119.9	N1—C9—H9B	109.8
C3—C2—H2	119.9	C8—C9—H9B	109.8
C4—C3—C2	119.48 (16)	H9A—C9—H9B	108.2
C4—C3—C7	119.94 (16)	C9—N1—H1A	111 (2)
C2—C3—C7	120.58 (16)	C9—N1—H1B	109 (2)
O1—C4—C5	117.33 (17)	H1A—N1—H1B	110 (3)
O1—C4—C3	122.96 (17)	C9—N1—H1C	112 (2)
C5—C4—C3	119.71 (17)	H1A—N1—H1C	102 (3)
C6—C5—C4	120.24 (18)	H1B—N1—H1C	113 (3)
C6—C5—H5	119.9	C4—O1—H1	106 (3)
C4—C5—H5	119.9	C7—O5—H5A	111 (2)
C5—C6—C1	120.18 (17)	C8—O7—H7	106 (2)
C5—C6—H6	119.9	O4—S1—O2	112.85 (11)
C1—C6—H6	119.9	O4—S1—O3	112.21 (10)
O6—C7—O5	122.70 (18)	O2—S1—O3	109.77 (10)
O6—C7—C3	123.42 (18)	O4—S1—C1	107.75 (8)
O5—C7—C3	113.88 (16)	O2—S1—C1	106.85 (9)
O8—C8—O7	125.65 (18)	O3—S1—C1	107.09 (9)
O8—C8—C9	122.73 (18)		
			
C6—C1—C2—C3	−0.8 (3)	C4—C3—C7—O6	−4.4 (3)
S1—C1—C2—C3	177.08 (14)	C2—C3—C7—O6	175.9 (2)
C1—C2—C3—C4	−0.2 (3)	C4—C3—C7—O5	175.01 (19)
C1—C2—C3—C7	179.42 (18)	C2—C3—C7—O5	−4.7 (3)
C2—C3—C4—O1	−179.32 (19)	O8—C8—C9—N1	11.5 (2)
C7—C3—C4—O1	1.0 (3)	O7—C8—C9—N1	−170.32 (16)
C2—C3—C4—C5	1.2 (3)	C2—C1—S1—O4	16.02 (18)
C7—C3—C4—C5	−178.49 (19)	C6—C1—S1—O4	−166.12 (16)
O1—C4—C5—C6	179.4 (2)	C2—C1—S1—O2	−105.50 (17)
C3—C4—C5—C6	−1.1 (3)	C6—C1—S1—O2	72.36 (17)
C4—C5—C6—C1	0.1 (3)	C2—C1—S1—O3	136.93 (15)
C2—C1—C6—C5	0.8 (3)	C6—C1—S1—O3	−45.21 (17)
S1—C1—C6—C5	−177.06 (16)		

### Hydrogen-bond geometry (Å, º)

**Table:**

*D*—H···*A*	*D*—H	H···*A*	*D*···*A*	*D*—H···*A*
O1—H1···O6	0.82 (1)	1.89 (2)	2.631 (2)	150 (4)
N1—H1*A*···O6	0.86 (1)	2.27 (3)	2.878 (2)	127 (3)
N1—H1*A*···O7^i^	0.86 (1)	2.46 (3)	3.134 (2)	135 (3)
N1—H1*B*···O3^ii^	0.87 (1)	2.06 (2)	2.876 (2)	157 (3)
N1—H1*C*···O3^iii^	0.87 (1)	1.98 (2)	2.811 (2)	161 (3)
O5—H5*A*···O4^iv^	0.82 (1)	1.92 (2)	2.702 (2)	159 (3)
O7—H7···O2^v^	0.82 (1)	1.84 (1)	2.646 (2)	169 (3)
C2—H2···O5^iv^	0.93	2.45	3.370 (2)	168
C9—H9*A*···O4^iv^	0.97	2.33	3.292 (2)	172
C6—H6···O8^vi^	0.93	2.46	3.273 (2)	147
C9—H9*B*···O2^vii^	0.97	2.37	3.324 (2)	167

Symmetry codes: (i) *x*−1, *y*, *z*; (ii) −*x*+1, *y*+1/2, −*z*+3/2; (iii) −*x*+1, −*y*+1, −*z*+2; (iv) −*x*+2, −*y*+1, −*z*+2; (v) −*x*+2, −*y*+1, −*z*+1; (vi) −*x*+1, −*y*+1, −*z*+1; (vii) −*x*+2, *y*+1/2, −*z*+3/2.
